# Gender Differentiated Preferences for a Community-Based Conservation Initiative

**DOI:** 10.1371/journal.pone.0152432

**Published:** 2016-03-29

**Authors:** Aidan Keane, Heather Gurd, Dickson Kaelo, Mohammed Y. Said, Jan de Leeuw, J. Marcus Rowcliffe, Katherine Homewood

**Affiliations:** 1 School of GeoSciences, University of Edinburgh, Edinburgh, United Kingdom; 2 Anthropology Department, University College London, London, United Kingdom; 3 ZSL Institute of Zoology, Regent’s Park, London, United Kingdom; 4 Kenya Wildlife Conservancies Association (KWCA), P.O Box 1038 000517, Nairobi, Kenya; 5 International Livestock Research Institute (ILRI), P.O. Box 30709 00100, Nairobi, Kenya; 6 The Center for Sustainable Drylands Ecosystems and Societies, University of Nairobi, P.O. Box 30297 00100, Nairobi, Kenya; 7 World Agroforestry Centre (ICRAF), P.O. Box 30677 00100, Nairobi, Kenya; Clemson University, UNITED STATES

## Abstract

Community-based conservation (CBC) aims to benefit local people as well as to achieve conservation goals, but has been criticised for taking a simplistic view of “community” and failing to recognise differences in the preferences and motivations of community members. We explore this heterogeneity in the context of Kenya’s conservancies, focussing on the livelihood preferences of men and women living adjacent to the Maasai Mara National Reserve. Using a discrete choice experiment we quantify the preferences of local community members for key components of their livelihoods and conservancy design, differentiating between men and women and existing conservancy members and non-members. While Maasai preference for pastoralism remains strong, non-livestock-based livelihood activities are also highly valued and there was substantial differentiation in preferences between individuals. Involvement with conservancies was generally perceived to be positive, but only if households were able to retain some land for other purposes. Women placed greater value on conservancy membership, but substantially less value on wage income, while existing conservancy members valued both conservancy membership and livestock more highly than did non-members. Our findings suggest that conservancies can make a positive contribution to livelihoods, but care must be taken to ensure that they do not unintentionally disadvantage any groups. We argue that conservation should pay greater attention to individual-level differences in preferences when designing interventions in order to achieve fairer and more sustainable outcomes for members of local communities.

## Introduction

Community-based conservation (CBC) aims to produce benefits for both conservation and economic development, often via mechanisms intended to influence local people's livelihood choices (e.g. the creation of new livelihood alternatives, payment schemes or information campaigns; [1]). CBC projects are increasingly widespread and much-advocated by conservation and government agencies [2], but there have been few high quality evaluations of their outcomes and evidence for their success is mixed [3]. Critics of these approaches have argued that they can fail to take account of the preferences and aspirations of the people whose behaviour they seek to alter and treat communities as if they are a single coherent entity, rather than a collection of heterogeneous individuals with differing motivations and preferences [4, 5]. The outcomes of such interventions are often characterised in simple terms (e.g. “win-win” for both conservation and development) without investigating in detail their effects on different groups within the target population [6, 7].

Previous research has frequently highlighted how the theoretical promise of CBC can be undermined by the realities of its implementation [8] and how benefits arising from CBC are commonly captured by small groups of elites, at the expense of the broader community (e.g. [9]). However, less attention has been paid to the subtler, but significant, trade-offs that can occur when an intervention is applied uniformly across groups of individuals holding different preferences and facing different opportunities and constraints. For example, in many of the areas where CBC is promoted males and females play markedly different roles in communities and occupy different places within power structures [10], meaning that the effect of CBC interventions on household livelihood portfolios can differentially affect men and women [4, 11]. Understanding the heterogeneity in preferences arising from such differences is important to highlight how conservation interventions might unintentionally favour or disadvantage specific groups, and to predict how people might behave in response [4, 6].

There is a long history of CBC throughout Africa, but in Kenya a new and important group of initiatives known as conservancies has emerged over recent years. Conservancies are defined by Kenya’s Wildlife Conservation and Management Act of 2013 as “Land set aside by an individual landowner, body corporate, group of owners or a community for purposes of wildlife conservation”. Since the first conservancies were established in the mid-1990s they have spread rapidly throughout the country and by 2013 there were an estimated 140 conservancies in existence. Conservancies differ in the details of their implementation, influenced by factors such as local land tenure arrangements. In the Mara, for example, where large areas of formerly communal land have been subdivided and privatised [12] the dominant model can be viewed as a form of payment for ecosystem services (PES) scheme which sees households lease plots of privately owned land to the conservancy to be used for ecotourism [13, 14]. The household forgoes some or all rights to reside on, cultivate or graze the land and in return they are paid an agreed rent per acre of land under conservancy management. By contrast, conservancies in northern Kenya, which together form the Northern Rangelands Trust, have tended to follow models based around communally-owned land with payments made to community organisations [15]. Despite their growing importance there have been few attempts to evaluate the outcomes of conservancy establishment (although see [14, 16, 17]), and there remains little understanding of how conservancy design can be tailored to local preferences or how they might affect different actors within a community.

In this study, we used discrete choice experiments to quantify the preferences of Maasai men and women around Kenya’s Maasai Mara National Reserve for different forms of livelihood and different levels of conservancy participation. Choice experiments are a stated-choice survey technique that is commonly used by economists to quantify the values of non-market goods and services, and to determine rates of substitution between them (i.e. how much of one thing an individual would be willing to give up to acquire one unit of another; [18]). They have a long history of application in the field of environmental economics [19] and recently there have been several examples of their use to address livelihood choices in conservation [20, 21]. In this context, a key advantage of the method is that it can be used to understand the relative importance of different components of an individual’s wellbeing.

Maasai livelihoods are customarily based around their livestock, with men and women playing markedly different cultural roles [22, 23]. Women are primarily responsible for day-to-day running of the household, milking the cattle, collecting water and firewood, preparing meals, caring for children and young or sick livestock; cultivating their fields and home gardens; building and maintaining their traditional houses [24]. Women rarely own cattle or land, though their husbands allocate milch cows for their use and sometimes land for them to farm, and women may own a few small stock. Women often sell milk in the market, and may run a petty trade, retailing small quantities of necessities like sugar from their homestead. Educated women based in urban areas may manage a shop, lodging house or other business for a relatively well-off husband. By contrast men generally own livestock and land and are responsible for their management, particularly herding cattle (with small stock often herded by children). Men manage livestock sales when cash is required for domestic needs, or for their own personal wants. Men spend considerable time consulting with their peers, manage interactions with government and outsiders such as tourism entrepreneurs, and take political and economic decisions on behalf of the household, including decisions around land use for grazing, for farming and/or for conservation set-aside [22, 23].

The different roles of men and women make it highly likely that any interventions which affect livelihoods in this area will differentially affect the two groups. We therefore set out to investigate the comparative values placed on different components of Maasai livelihoods and to test whether men and women differ in their preferences for different types of livelihood. Because conservancies were already in existence in the area when our study was carried out, we also investigated whether there are differences between the preferences of existing conservancy members and non-members. Finally, we examined the extent to which any differences can be attributed to differences in the distribution of other characteristics (e.g. wealth, education) between the sexes and between members and non-members. Although the latter relationships are of interest in their own right, for the purposes of this analysis we treat these additional characteristics primarily as nuisance variables.

## Materials and Methods

### Study area

**Fig 1:**
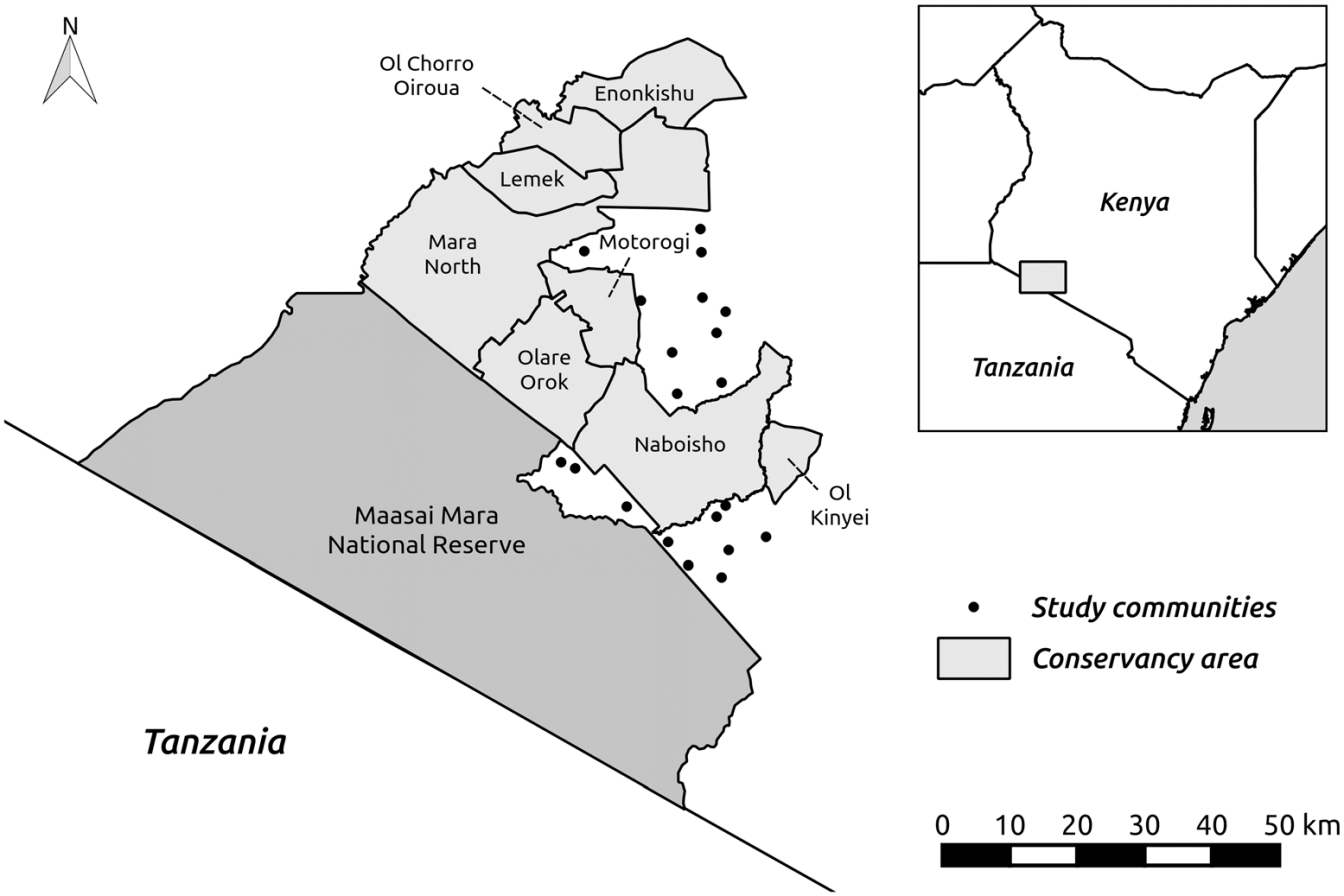
Map of our study locations. The map shows the boundaries of the conservancies in our study area and the approximate location of communities whose members participated in our choice experiments.

We conducted our study across 20 villages in Narok County, Kenya (Fig 1). At the time of data collection there were eight active conservancies in the area, managing a total of 92,248 ha of land. Our survey areas were adjacent to the Maasai Mara National Reserve, Naboisho, Olare Orok & Motorogi and Mara North conservancies and included land within the former Koiyaki Group Ranch (which was subdivided and allocated to private ownership in 2003–2004) and Siana Group Ranch. This area is famous for its large mammal populations, forms the northern boundary of the Serengeti-Mara Ecosystem [25] and generates substantial tourism revenues, with the Maasai Mara National Reserve being Kenya’s most profitable protected area [26]. However, prior to the creation of conservancies relatively little of the money was retained in the areas closest to the reserve and many households remain poor [27].

Although the cornerstone of Maasai livelihoods is their livestock, the majority of households also pursue other activities including the small-scale cultivation of crops such as maize, wheat and beans, and wage-earning activities such as petty trading, small businesses and employment by government, NGO or private enterprises [23]. Alongside this, the establishment of conservancies has created new opportunities for landowners to lease land for ecotourism. The key livelihood decisions that local people make therefore involve trade-offs in the allocation of land, labour and financial resources between these competing opportunities.

### Choice experiment design

Participants were asked to make a series of choices between pairs of alternative livelihood scenarios (e.g. Table 1; see also S1 Appendix, for further details about our experimental design). Each alternative was created from combinations of six attributes and each attribute could take on either two or three distinct levels (Table 2). The alternatives were not named or otherwise labelled, so differed only in terms of these attributes. In designing the experiment we drew upon a combination of existing information, expert opinion and pre-testing. Initial scoping of the relevant attributes for the choice task and specification of levels for these attributes was based on a review of the substantial body of existing literature which has examined livelihoods and conservation in the Maasai Mara area (e.g. [23, 28]) and the experience of DK and KH. This preliminary design was refined based on discussions with local key informants and piloted with a small group of respondents who performed the choice tasks and were subsequently debriefed for their experiences. The discussions aimed to explore whether the scenarios presented were relevant and plausible, whether the attributes levels were realistic, whether the verbal presentation of choice situations was acceptable and whether respondents were able to maintain concentration. The combination of attribute levels within the choice tasks was chosen to follow a blocked fractional factorial design [18] created using the AlgDesign package in R [29]. The result was an experimental design comprising 16 distinct choice situations. To help participants to maintain concentration, each participant was only presented with eight choice situations: half of the respondents received the first block of eight, and half received the second block of eight. The blocking was carried out as part of the design phase using the function optBlock, which aims to minimise D-error [29]. The D-error of the optimal design was 0.221.

**Table 1:** An example of one of the choice situations presented to participants.

	Option 1	Option 2
*Number of cattle*	100	40
*Number of sheep or goats*	0	0
*Private grazing land and monthly conservancy payments*	No private grazing land, 18,000 KSh conservancy payments	75 acres private grazing land, 9,000 KSh conservancy payments
*Grazing permitted in conservancy during drought*?	No	No
*Monthly wage*	6,000 KSh	6,000 KSh
*Area of land cultivated*	0	5 acres

This choice situation is one of sixteen which formed the basis of a discrete choice experiment intended to examine the participants’ preferences for livelihoods and conservation. The participants were members of local communities living near to the Maasai Mara National Reserve, Kenya, and included both members and non-members of existing conservancies. The participant simply responds by saying whether they would prefer option 1 or option 2.

**Table 2:** Attributes used in the choice experiment design.

Attribute	Levels	Coding	Description
*Access*	1. Allowed; 2. Prohibited	Categorical	Whether or not the conservancy allows grazing access to conservancy land during times of drought.
*Cattle*	1. 0 animals; 2. 40 animals; 3. 100 animals	Continuous	Number of cattle owned by the household.
*Conservancy involvement*	1. None; 2. 50% of household land for 9,000 KSh; 3. 100% of household land for 18,000 KSh	Categorical	The proportion of the household’s land that is leased to the conservancy and the resulting monthly payment received. For consistency, participants were asked to imagine a scenario in which their household owned 150 acres of land in total.
*Cultivation*	1. None; 2. 5 acres	Categorical	Whether or not the household practiced subsistence cultivation.
*Small stock*	1. 0 animals; 2. 80 animals; 3. 200 animals	Continuous	The number of small stock owned by the household.
*Wages*	1. 0 KSh; 2. 6,000 KSh; 3. 10,000 KSh	Continuous	The monthly cash income received by members of the household for wage-earning activities.

Description of the attributes and levels used in the design of the choice experiment, along with information about their coding for the purposes of statistical analysis.

### Survey administration

Field work was carried out by HG, DK and two field assistants. Both DK and the field assistants are local to the area and speak Maa and Swahili fluently. Simple random sampling of respondents within our study area was not possible because there were no complete village registers available for use as a sampling frame and access to participants had to be granted by community leaders. We therefore adopted a form of cluster sampling. First we sampled villages from a full list of settlements within the study area then, within each village, we aimed to speak to ten men and ten women. A total of 388 respondents participated in the study, completing the choice experiments between 31st January and 19th June 2013. Participants were read a standard introductory text (S2 Appendix). In addition to recording their choices, we collected data on several socio-economic variables: characteristics of the participant (sex; age; age set [22, 30]; whether they were married; whether they had children; whether they had received any formal education; and their primary occupation), characteristics of their home (location; number of years living at this location; number of buildings; number of buildings with corrugated iron roof), whether they were members of a conservancy, whether they occupied positions of authority (head of the household; leadership positions within community), the quantity of livestock and land owned by the household (cattle; small stock, total area of land, land within a conservancy), and whether any vehicles were owned by the household (see S1 Table).

### Ethics statement

This research was approved by the ethics committee of the Department of Anthropology, University College London. We were careful to obtain the free, informed consent of all subjects prior to their participation in the study and participants were told that they were free to withdraw their consent at any point if they so wished. Because levels of literacy are very low in the study area, consent was obtained verbally and recorded by the field team prior to data collection. If consent was not granted, no further information was requested. This consent procedure was approved by the ethics committee. The choice data were anonymised prior to digitisation and no information that would allow individual participants to be identified is presented in this paper.

### Statistical analysis

Statistical analyses were carried out in R version 3.1.3 [31] and Stan [32], via the RStan package [33]. The choice data were modelled using Bayesian hierarchical generalised linear models with binomial errors. This is equivalent to a mixed logit model (also commonly called a random parameters logit model) with two alternatives. The response was a binary variable that indicated whether the respondent preferred the first or second hypothetical alternative in each choice set. Predictor variables included: (1) the comparative attributes of the two choice items (e.g. the difference in number of cattle between the available choices) which vary from choice situation to choice situation, and (2) the socio-demographic characteristics of the respondent (i.e. sex, age, education) which vary between individuals. Models also included an intercept term to model any remaining, unexplained tendency to choose the second option. Choice attribute effects were allowed to vary between individuals, modelling individual heterogeneity in preferences while accounting for the panel structure of the data in which each respondent made several choices. Since individual-specific characteristics do not vary from choice to choice they enter the model as interactions with the attributes of the choice item, describing how preferences for these attributes vary between groups of individuals [18]. Socio-economic characteristics were therefore modelled as hierarchical, individual-level predictors of the choice-level attribute effects. For further details of the statistical analysis please refer to S3 Appendix.

We fitted three variants of this model. The first model (hereafter, Model 1) included only choice attributes as predictors, providing estimates of the average value of each livelihood component across our entire sample. The second model (Model 2) added sex and conservancy membership as individual-level predictors, corresponding to the key elements of differentiation that we set out to investigate. The final model (Model 3) included all available socio-economic characteristics of the participants as predictors, to investigate whether any differences observed between sexes, wealth groups and conservancy members and non-members were linked to other socio-demographic differences between these groups. In the text we report means and 95% credible intervals (hereafter, 95% CI) of posterior distributions derived from the models. Previous studies using choice experiments have often presented results in the form of estimates of “willingness to pay” (i.e. the average rate of substitution between a monetary choice attribute and other attributes; [18]), but a multi-site study of more than 1,000 households across five sites in Kenya and Tanzania concluded that “[l]ivestock holdings represent the single strongest measure or indicator of other dimensions of wealth and income in all sites” ([23], Ch. 10). Here, we therefore focus primarily on the rates of substitution between cattle and other choice attributes since cattle are a more meaningful and culturally appropriate common currency for comparing preferences in this area.

## Results

### What value do Maasai pastoralists place on different components of their livelihoods?

As expected, the average respondent’s utility was increased by higher wages, more cattle or small stock, successful cultivation, having access to the conservancy land for grazing during drought, and leasing land to the conservancy at current levels of conservancy payments (Table 3). Cattle were valued approximately twice as much as small stock, with one sheep or goat equivalent to 0.54 head of cattle (95% CI: 0.43, 0.65). Access to conservancy land for dry season grazing was highly valued, equivalent to a herd of 60.08 cattle (95% CI: 48.21, 73.63) while successful cultivation was equivalent to 44.50 cattle (95% CI: 34.44, 55.22). Interestingly, while participants placed considerable value on leasing half of their land to a conservancy, equivalent to a herd of 86.03 cattle (95% CI: 67.69, 105.04), leasing all of their land was seen as far less beneficial, equivalent to only 9.02 cattle (95% CI: -8.24, 27.77) on average.

**Table 3:** Parameter estimates from Model 1.

	Posterior mean	Std. dev.
*Parameter*	*Estimate (95% CI)*	*Estimate (95% CI)*
Intercept	-0.20 (-0.31, -0.09)	0.18 (0.00, 0.33)
Access (Yes)	1.11 (0.90, 1.32)	0.29 (0.00, 0.63)
Cattle (100 head)	1.86 (1.52, 2.22)	1.79 (1.38, 2.28)
Conservancy (150 acres)	0.16 (-0.15, 0.49)	0.96 (0.29, 1.57)
Conservancy (75 acres)	1.59 (1.33, 1.86)	1.28 (0.96, 1.59)
Cultivated (5 acres)	0.83 (0.60, 1.05)	0.64 (0.32, 0.99)
Small stock (200 head)	1.99 (1.53, 2.45)	2.12 (1.57, 2.67)
Wage (10,000KSh/month)	0.48 (0.23, 0.72)	0.29 (0.00, 0.65)

Parameter estimates from Model 1, a mixed logit model with only choice attributes (i.e. the experimentally manipulated characteristics which defined each choice situation, e.g. number of cattle; Table 2) included as predictor variables.

Model 1 also revealed substantial heterogeneity in preferences. The greatest variation between individuals was found for the values placed on small stock and cattle, leasing land to a conservancy and cultivation (Table 3). In the case of conservancy membership, 42.51% of individuals (95% CI: 23.71, 58.51) actually place a negative value on leasing all of their land to a conservancy. As would be expected, the values placed on certain predictors were strongly correlated with one another. In particular, individuals who placed greater value on cattle also tended to place greater value on small stock (correlation, ρ = 0.85; 95% CI: 0.70, 0.95), cultivation (ρ = 0.64; 95% CI: 0.31, 0.87) and having 50% of household land within a conservancy (ρ = 0.57; 95% CI: 0.29, 0.81).

### Does the value placed on livelihood components differ by sex and conservancy membership?

When interactions between choice attributes and the sex and conservancy membership status of the participant were modelled (Model 2), the average male non-member placed a positive value on livestock, wage income, successful cultivation and access to grazing within the conservancy (Table 4). Having half of their land within a conservancy was also seen as positive, equivalent in value to 45.92 cattle (95% CI: 10.35, 85.65), but in this group having all land within a conservancy was unambiguously negative, equivalent to the loss of 43.29 cattle (95% CI: -81.18, -6.18).

**Table 4:** Parameter estimates from Model 2.

	Posterior mean: Male non-members	Posterior mean: Female non-members	Posterior mean: Male conservancy members
*Parameter*	*Estimate (95% CI)*	*Estimate (95% CI)*	*Estimate (95% CI)*
Intercept	-0.14 (-0.31, 0.04)	-0.48 (-0.69, -0.26)	0.04 (-0.16, 0.24)
Access (Yes)	1.26 (0.92, 1.60)	0.52 (0.12, 0.91)	1.70 (1.33, 2.07)
Cattle (100 head)	1.24 (0.69, 1.79)	1.70 (1.02, 2.36)	2.04 (1.45, 2.67)
Conservancy (150 acres)	-0.54 (-1.04, -0.02)	0.02 (-0.54, 0.65)	0.15 (-0.40, 0.74)
Conservancy (75 acres)	0.54 (0.19, 0.90)	1.13 (0.69, 1.58)	1.89 (1.48, 2.35)
Cultivated (5 acres)	0.95 (0.59, 1.37)	0.53 (0.12, 0.98)	1.16 (0.77, 1.60)
Small stock (200 head)	1.54 (0.87, 2.24)	1.57 (0.73, 2.44)	2.33 (1.56, 3.14)
Wage (10,000 KSh/month)	0.91 (0.51, 1.30)	0.08 (-0.34, 0.53)	0.92 (0.48, 1.35)

Combined parameter estimates from Model 2, a mixed logit model including interactions between the choice attributes and dummy variables indicating the gender of the respondent and conservancy membership status.

The preferences of female respondents differed from those of males in several respects. Women placed greater value on conservancy membership, but less value on cultivation and access to conservancy land for grazing. They also placed substantially less value on wage income: while the average male non-member values a monthly salary of 10,000 KSh equivalent to a herd of 76.75 cattle (95% CI: 36.26, 122.11), the average female non-member values the same salary at 4.67 cattle (95% CI: -23.47, 31.98).

Conservancy members placed greater value on livestock, having some or all of their privately owned land within a conservancy and having access to grazing land within the conservancy than non-members. Thus, for example, the average male conservancy member placed a small positive value on having all of their privately owned land within a conservancy, equivalent to 8.81 cattle (95% CI: -19.90, 39.88), but a much larger value on having half of their land within a conservancy, equivalent to 94.45 cattle (95% CI: 64.67, 131.88).

### Can differences in values be explained by other socio-economic differences between these groups?

**Fig 2:**
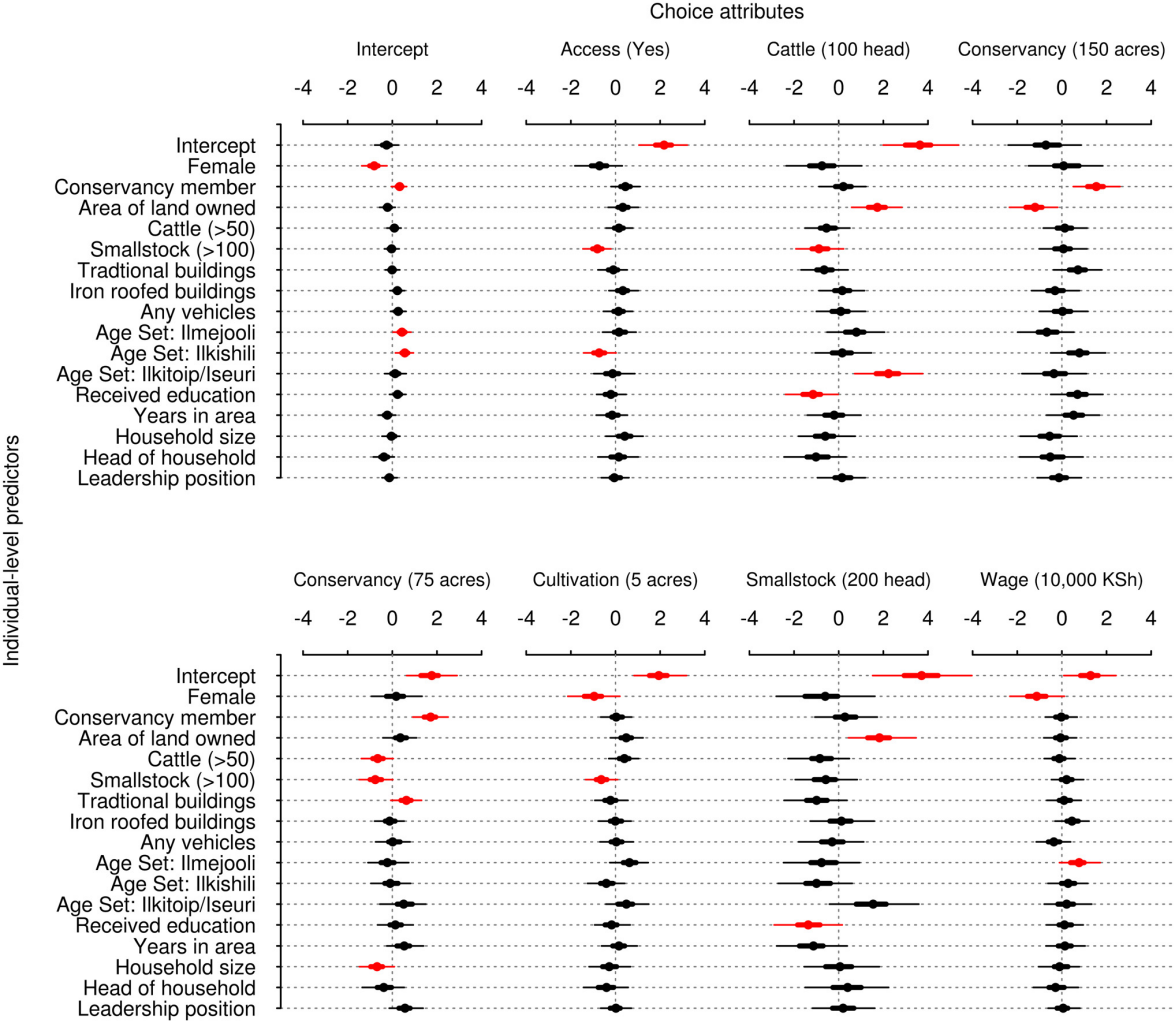
Parameter estimates from Model 3, the fitted model including the full suite of interactions between socio-economic characteristics and choice attributes. Points indicate posterior means, thin lines indicate 95% highest posterior density interval and heavy lines indicate 50% highest posterior density intervals. As a visual aid to interpretation, parameters whose 90% highest posterior density intervals do not include zero are highlighted in red.

The final model, including the full suite of individual characteristics (Model 3), suggests that some of the observed differences between males and females, conservancy members and non-members, are linked to the distributions of other socio-economic characteristics within these groups (Fig 2).

There were clear differences in preferences associated with indicators of wealth. For example, respondents who owned a larger area of land tended to strongly prefer cattle and small stock, and placed less value on having all of their land within a conservancy. Those owning larger numbers of cattle or small stock placed less value on having half of their land in a conservancy. Those with more small stock also placed less value on cultivation and, perhaps surprisingly, on having access to grazing on conservancy land and on owning cattle. Respondents who owned a greater number of traditionally constructed buildings were slightly more favourable towards conservancy involvement, but there were no clear effects associated with ownership of iron-roofed buildings or vehicles.

This model also reveals differences between different age groups, with the oldest age sets (Ilkitoip and Iseuri) placing substantially greater value on cattle than the younger age sets, while the Ilkishili and Ilmejooli age sets placed marginally less value on access to grazing within conservancy lands and on wage income respectively. Participants who had received formal education placed less value on both cattle and small stock while those from larger households placed less value on having half of their land within a conservancy. There were no clear effects of being household head, of having lived in the area for longer or of holding a community leadership position on preferences for any of the livelihood components.

Having accounted for these effects, however, certain differences remained between the sexes and conservancy members and non-members: female participants still placed less value on wage earnings and on cultivation than did comparable male respondents, and conservancy members placed greater value on land within a conservancy than did comparable non-members.

## Discussion

Our results demonstrate how preference heterogeneity could lead to particular groups within communities, such as men and women, experiencing different outcomes from a conservation intervention [4] and make an important contribution to the debate around the design of fair and effective conservation interventions with broader relevance for CBC across the Global South. For the specific case of the Mara conservancies, our findings also provide new insight into current Maasai livelihood preferences following the rapid growth of community-based conservancy initiatives throughout Kenya.

The customary prominence of livestock in Maasai life and culture can be seen in our results in the high value placed on cattle, small stock and having access to conservancy land for grazing. The latter may have been made more salient by ongoing discussions about grazing on conservancy land, but likely reflect a genuine assessment of the importance of grazing reserves as an emergency resource to be used in times of drought to supplement or replace traditional long distance migration in an increasingly fragmented landscape [34]. Previous research in conservancies in the Northern Rangelands Trust suggests that allowing dry season grazing within the areas under conservancy management has been key to their acceptance by local people [16]. By contrast, the decision to exclude grazing from Tanzania’s Burunge Wildlife Management Area has led to ongoing conflict [35].

Despite the continuing importance of livestock, however, Maasai livelihood strategies are diverse and responsive to changes in political and economic circumstance [23, 36–38]. This preference for diversified pastoralist livelihoods was clearly reflected in our results, with both cultivation and wage labour being positively valued alongside livestock and grazing. Maasai willingness to incorporate new components into their livelihoods also extends to participation in conservation initiatives [38]. Conservancies are a relatively novel development in the Maasai Mara region, with the first having been established in 2006, but on average our participants placed a positive value on conservancy membership so long as they were able to retain land outside of conservancy management for grazing or settlement. By contrast, it is clear that many would not wish to give over all of their land to conservancies at current levels of payments, if at all.

Underlying these mean preferences, we found evidence of substantial individual heterogeneity and important differences between groups within the community. For example, women valued conservancy membership more highly than men did, but placed little value on cash obtained through wage labour. There is an apparent contradiction here in that conservancy membership involves giving up rights to land in return for cash payments, but the difference may lie in the amount of control women are able to exert in each case. Wages are primarily earned by men and often contribute little to household budgets [39]. By contrast, the Mara conservancies pay fees directly to households’ bank accounts, and have helped members to open new accounts where necessary [14]. Although these accounts are registered in the name, and entirely under the control, of heads of household (who are predominately male), the regular monthly conservancy payments may be more visible to, and open to influence by, women. In other areas of Kenya the timing of conservation payments is deliberately targeted towards meeting household needs. For example, PES payments in Kitengela coincide with the dates when school fees are due, to encourage investment in education [40]. Alternatively, women’s relative preference for conservancy membership could also be influenced by the community-level benefits of conservancies that were not disaggregated in our choice experiments (e.g. infrastructure, health facilities, roads).

Other individual characteristics were also associated with notable differences in livelihood preferences. For example, existing conservancy members place greater value on having land within a conservancy than do non-members. Although this conforms to our expectations, we cannot tell from our data whether it reflects a form of self-selection (e.g. those who were positive towards conservancies are more likely to have become members) or a change of preferences caused by positive experience of conservancy membership (e.g. members forming stronger preferences for conservancies having experienced benefits). On average conservancy members also valued livestock and the ability to graze on conservancy land more highly than did non-members, but these differences appear to be linked to the distributions of other socio-economic characteristics between the two groups, and largely disappear when they are controlled for. For example, conservancy members own more land than non-members and a higher proportion belong to the oldest age set and we found that both characteristics are linked to stronger preferences for cattle ownership. By contrast, younger participants and those who had received more education were found to place less value on cattle, perhaps reflecting changing expectations and aspirations [41].

Participants who owned more cattle and small stock were understandably less favourable towards conservancy involvement, as set-aside places greater pressure on their ability to find grazing for their livestock [42]. More surprising was the fact that those with more small stock placed less value on cattle and on access to grazing land within conservancies. Alongside the relatively high value placed on small stock relative to cattle, this may reflect an ongoing change in livelihood strategies away from traditional extensive grazing. Independent observations suggest that a shift from cattle to small stock is occurring throughout the region [43]. Small stock may be easier to accommodate in an increasingly fragmented landscape, particularly the popular Dorper sheep which are seen as easily managed, although less drought resistant [44]. Shifting market demand may also mean small stock are attracting disproportionately profitable prices.

Our findings have several implications for conservation policy. Within our study area, the strategy pursued by conservancies assumes that loss of grazing land can readily be compensated by cash payments, but our results do not bear this out. Despite the fact that our choice scenarios offered a constant level of conservancy payments per hectare enrolled, having all of a household’s land under conservancy management was seen as negative by large proportion of the respondents, and the majority of participants valued this less than having half of their available land within a conservancy. As previously noted, the conservancy model adopted in the Mara can be viewed as a form of PES, and the lack of straightforward equivalence between cash payments and other livelihood-related resources presents an important challenge to the success and sustainability of many PES schemes [45].

The model followed by the Maasai Mara conservancies also implicitly assumes that loss of grazing land and provision of payments will lead people to move away from livestock grazing. However, our findings indicate that the Maasai retain strong preferences for livestock, and these preferences were in fact stronger amongst the conservancy members in our sample than among equivalent non-members. Rather than shift away from pastoralism, Butt [46] suggests that richer households, who are in a position to pay cash fines if caught, adopt a strategy of grazing their livestock illegally within the Mara reserve in order to maintain their herds. Conservancies that exclude grazing could therefore increase pressure on adjacent protected areas and heighten the risk of conflict (cf. [35]). These findings about the importance of maintaining grazing and livestock alongside conservancies also present useful lessons for Tanzania’s WMAs which are conceptually similar to Kenya’s northern conservancies, but with the state acting as a middleman, regulator and prime beneficiary in the interactions between communities and investors.

Finally, by highlighting the existence of multiple heterogeneous preferences within a “community”, our results also serve to demonstrate how well-meaning conservation interventions can unintentionally disadvantage some groups relative to others if insufficient attention is paid to the social structure and politics of the communities they seek to engage [4]. For example, although the women in our study appear to value conservancies more than men do on average, they placed very little value on wage income. Therefore, if the loss of grazing land to conservancies was to shift livelihoods away from livestock and towards wage labour, this may have unexpected negative consequences for women. The differential effects of interventions on men and women has parallels in the community-based development literature (e.g. loss of access to resources by women after enclosure of commons in India and tree planting in Gambia; [47, 48]), but remains an under-researched topic in conservation with important implications for the just and equitable design of PES and CBC initiatives [49].

Although CBC initiatives are an established feature of the conservation landscape, there remains much to be learned about how conservation organisations can work with communities in ways that are mutually beneficial and do not unfairly disadvantage any groups or individuals, particularly those who are least able to represent their own interests. By focussing attention on the importance of the heterogeneity in preferences and motivations that exists between individuals, we hope that this work will contribute to a richer, differentiated understanding of community in conservation.

## Supporting Information

S1 Appendix Further details of the experimental design and implementation. (DOCX)

S2 Appendix Instructions for participants in the discrete choice experiments. (DOCX)

S3 Appendix Additional details of the statistical analysis. (DOCX)

S1 Data Data about choices. The data are recorded with one observation per row and one variable per column. The variables are coded as follows: choice = dummy variable indicating whether the first option (0) or second option (1) was chosen; access = difference between two dummy variables indicating whether access for grazing is allowed in the first and second options (-1; 0; 1); cattle = difference in the number of cattle in the first and second options (-100; -60; -40; 0; 40; 60; 100); cons150 = difference between two dummy variables indicating whether all of an individual’s private land is under conservancy management or not in the first and second options (-1; 0; 1); cons75 = difference between two dummy variables indicating whether half of an individual’s private land is under conservancy management or not in the first and second options (-1; 0; 1); cult = difference between two dummy variables indicating whether cultivation takes place in the first and second options (-1; 0; 1); smlstk = difference in the number of small stock in the first and second options (-200; -120; -80; 0; 80; 120; 200); wage = difference in the monthly wage earned in the first and second options (-10,000; -6,000; -4,000; 0; 4,000; 6,000, 10,000); indiv_id = unique identifier for each respondent (1 to 388); choice_id = unique identifier for each choice situation (1 to 16).
(CSV)

S2 Data Data about individual characteristics. The data are recorded with one observation per row and one variable per column. The variables are coded as follows: indiv_id = unique identifier for each respondent (1 to 388); sexF = dummy variable indicating whether respondent was female (1) or male (0); consY = dummy variable indicating whether respondent was a conservancy member (1) or not (0); land = area of land owned by the respondent (acres); cattle51plus = dummy variable indicating whether respondent owned more than 51 head of cattle (1) or not (0); smlstk101plus = dummy variable indicating whether respondent owned more than 101 head of small stock (1) or not (0); buildingsMud = number of traditional buildings owned by respondent; buildingsIron = number of iron-roofed buildings owned by respondent; vehicle = dummy variable indicating whether respondent owned one or more vehicles (1) or not (0); ageset2 = dummy variable indicating whether respondent belonged to the Ilmejooli age set (1) or not (0); ageset3 = dummy variable indicating whether respondent belonged to the Ilkishili age set (1) or not (0); ageset4 = dummy variable indicating whether respondent belonged to the Ilkitoip or Iseuri age sets (1) or not (0); educated = dummy variable indicating whether the respondent had received any formal education (1) or not (0); settlementYears = the length of time that the respondent had lived in the area (years); hhsize = household size (number of people); olmareiHead = dummy variable indicating whether the respondent was the head of the household (1) or not (0); leadership = dummy variable indicating whether the respondent held any leadership positions (1) or not (0).
(CSV)

S1 Table Summary of socio-economic characteristics of participants, grouped by sex and conservancy membership. Age sets are groupings of individuals of similar ages with distinct social and cultural roles and responsibilities (see [22, 30] for a full description). Within our sample the age ranges of the men in each age set were: Ilmeshuki, 18–38; Ilmejooli, 23–45; Ilkishili, 35–52; Ilkitoip/Iseuri, 48–74. Married women take on the age set of their husbands, so are commonly younger than their age set would suggest. The age ranges for women associated with each age set were: Ilmeshuki, 16–32; Ilmejooli, 16–38; Ilkishili, 18–45; Ilkitoip/Iseuri, 16–70.
(DOCX)
