# Supplementary material for: Gender Differentiated Preferences for a Community-Based Conservation Initiative
Source: PLoS One. 2016 Mar 29;11(3):e0152432. doi: 10.1371/journal.pone.0152432 (PMC4811562; doi:10.1371/journal.pone.0152432)
Supplement: S1 Appendix — (DOCX) [file pone.0152432.s001.docx]

*Design of choice task*

Although status quo alternatives are commonly used, it is important that choice experiments are designed in a way which mimics real life insofar as possible (Hanley et al. 2001; Hoyos 2010; Reed Johnson et al. 2013). We therefore made the decision not to include one in our design as this better reflects the reality of the situation in the Maasai Mara, where the study population have experienced a series of changes that have forced them to choose between novel alternatives with no possibility for “no change” to their livelihoods (Homewood et al. 2009; Thompson et al. 2009). The key theoretical argument for the inclusion of a status quo option is that, in order for the results to be consistent with demand theory and interpretable in welfare economic terms, at least one of the options must be in the respondents' feasible choice set (e.g. Hanley et al. 2001). We were therefore careful to ensure that all of the alternatives presented were composed of combinations of attributes that are currently represented within the study area or could realistically be introduced at the present time.

*Presentation of the choice task*

Levels of literacy are low in our study area, so the choice tasks were read out to participants. Illiteracy poses important practical challenges for the design and implementation of choice experiments. In our specific case the Maasai have a strong oral tradition (Kipury 1983) and are comfortable with communicating and receiving information in spoken form (as well as challenging and debating it) where the appropriate customary forum and processes are observed (Goldman & Milliary 2014). Several aspects of our choice experiment design were specifically chosen in order to limit the difficulty of the choice task, including the decisions to provide only two alternatives per choice situation and that each alternative should be defined by only six attributes that would already be very familiar to respondents. We also explained to respondents that we would repeat the information as many times as they desired (see Supporting Information, S2_File.docx). Our pilot study suggested that these strategies were effective and that respondents were able to retain, consider and act upon the information needed to make each choice so we are confident that this did not introduce any undue bias into our data.

Goldman, M., and S. Milliary. 2014. From critique to engagement: re-evaluating the participatory model with Maasai in Northern Tanzania. Journal of Political Ecology **21**:408-423.

Hanley, N., S. Mourato, and R. E. Wright. 2001. Choice Modelling Approaches: A Superior Alternative for Environmental Valuatioin? Journal of Economic Surveys **15**:435-462.

Homewood, K., P. Kristjanson, and P. Chenevix Trench, editors. 2009. Staying Maasai? Livelihoods, Conservation and Development in East African Rangelands. Springer New York, New York.

Hoyos, D. 2010. The state of the art of environmental valuation with discrete choice experiments. Ecological Economics **69**:1595-1603.

Kipury, N. 1983. Oral literature of the Maasai. Heinemann Educational Books.

Reed Johnson, F., E. Lancsar, D. Marshall, V. Kilambi, A. Mühlbacher, D. A. Regier, B. W. Bresnahan, B. Kanninen, and J. F. P. Bridges. 2013. Constructing Experimental Designs for Discrete-Choice Experiments: Report of the ISPOR Conjoint Analysis Experimental Design Good Research Practices Task Force. Value in Health **16**:3-13.

Thompson, D. M., S. Serneels, D. Ole Kaelo, and P. Chenevix Trench. 2009. Maasai Mara – Land Privatization and Wildlife Decline: Can Conservation Pay Its Way? in K. Homewood, P. Kristjanson, and P. Chenevix Trench, editors. Staying Maasai? Livelihoods, Conservation and Development in East African Rangelands. Springer New York, New York.
