# Supplementary material for: Gender Differentiated Preferences for a Community-Based Conservation Initiative
Source: PLoS One. 2016 Mar 29;11(3):e0152432. doi: 10.1371/journal.pone.0152432 (PMC4811562; doi:10.1371/journal.pone.0152432)
Supplement: S2 Appendix — (DOCX) [file pone.0152432.s002.docx]

**Instructions for participants in the discrete choice experiments**

NB Notes to the research assistant are marked in *italics.*

- In this exercise you will be presented with a series of eight cards which all look something like this [*show a blank card*].
- Each of these eight cards will describe two different hypothetical (imaginary) scenarios [*point at options on card*]; option 1 [*highlight whole of column 1*] and option 2 [*highlight whole of column 2*].
- Each option is described by the same set of characteristics [*point at attributes*]. There are six characteristics in total; all relate to possible livelihood opportunities in this region.
- For each of the eight cards we will read out the details corresponding to option 1 followed by the details corresponding to option 2.
- We would like you to imagine yourself in one year’s time and decide which of these options - based on the information provided - you would prefer if you had to make a choice between the two. Although these choices are imaginary, they are designed to reflect situations that could occur this area. We’d like you to think carefully about your choices and imagine how you would feel if these changes really happened.
- We will go through the six characteristics now so you know what to expect. Please ask questions if anything is unclear.
- The six characteristics we will us to describe each option are:

1. **The number of cattle owned**

[0 / 40 / 100]

1. **The number of sheep and goats (shoats) owned**

[0 / 80 / 200]

1. **The amount of private grazing land available which is in turn linked to the amount of land available to lease to conservancies.**

[150 acres + 0KSh / 75 acres + 9,000KSh / 0 acres + 18,000KSh]

[*Explain in terms of owning 150 acres of land and the three options available would be (a) all 150 acres as private grazing and none leased to conservancy; (b) spilt 50:50 between private grazing and conservancy, so conservancy payment based on 75 acres is 9,000KSh and (c) all 150 acres leased to conservancy, so conservancy payment is 18,000KSh but no land left for private grazing.*]

1. **Whether or not grazing is allowed inside a conservancy during drought**

[Yes / No].

1. **Monthly income from waged employment**

[0KSh / 6000KSh / 10000KSh]

1. **Area of land cultivated**

[0 acres / 5 acres]

- Option 1 and option 2 will differ only in the values associated with each characteristic.
- There will be eight cards in total so eight choices to be made.
- To reiterate, please imagine yourself one year from now and decide whether you would prefer to be in the situation described by option 1 or the situation described by option 2. Please think carefully about these choices.
- We will of course repeat any information you need to hear again in order to make each decision.

The idea is that by repeating this exercise with lots of different individuals it will help us build a better understanding of livelihood preferences in this region. So, of the livelihood opportunities that are available, which are valued more highly and which are not valued so highly? You can of course refuse to answer any or all of the questions. However, the individual answers you provide will be kept strictly private and not disclosed to others. As we said before, the purpose of the exercise is to help us understand your livelihood preferences as a whole. Since we will be repeating this many other groups we ask that you do not discuss the choices you made or any other information about the exercise with others. Different sets of cards will be presented to different people.

**Are there any questions?**
