# Supplementary material for: Gender Differentiated Preferences for a Community-Based Conservation Initiative
Source: PLoS One. 2016 Mar 29;11(3):e0152432. doi: 10.1371/journal.pone.0152432 (PMC4811562; doi:10.1371/journal.pone.0152432)
Supplement: S3 Appendix — (DOCX) [file pone.0152432.s003.docx]

*Modelling approach*

The standard statistical framework for the analysis of discrete choice experiments is based on the multinomial logit model (e.g. Hoyos 2010; Louviere et al. 2000). We use a version of the multinomial logit, although in our case the respondents were required to choose between two alternatives in each choice situation. In this case, the appropriate response distribution is a multinomial with two categories, which is more commonly referred to as the binomial distribution. This model is equivalent to a binomial generalised linear model in which the predictor variables are coded as the differences between the values of the predictors for the two alternatives (e.g. Aizaki et al. 2014 p.79; see also Nielsen et al. 2014 for a recently published study which has also formulated the model in this way).

A common extension of the basic multinomial logit framework allows for batches of parameters to be modelled as random draws from a shared probability distribution (Louviere et al. 2000). This is often referred to as a mixed logit model or random parameters logit model. We adopt this approach, fitting a model in which all of the parameters corresponding to choice attributes to vary between individuals (to model individual differences in preferences), and include individual characteristics as "group level predictors" which allows the individual-specific parameters to be estimated more precisely (Gelman et al. 2014).

*Data preparation*

Within our models the variables representing the numbers of cattle and small stock and value of monthly wage choice attributes were represented as continuous variables while access to the conservancy for grazing, amount of land leased to the conservancy and area cultivated were modelled as categorical variables using dummy coding (Table 1 in the main text). Prior to modelling, continuous variables corresponding to choice attributes were centred and scaled to lie on the range -1 to +1, while those corresponding to socio-economic characteristics were centred and scaled by subtracting their means and diving by two standard deviations prior to analysis (Gelman 2008). Credible intervals for quantities of interest were derived from the MCMC samples as highest posterior density intervals using the coda package in R (Plummer et al. 2006).

*Prior distributions*

We chose to use weakly informative priors centred on zero, with variances which reflected our prior expectations about the plausible range of values that the parameters might take (Gelman 2008). Weakly informative priors serve to regularize the posterior distributions of the parameters which in turn reduces the risk of overfitting the data (Gelman et al. 2014). The parameters for choice attributes and individual characteristics were assigned weakly informative normal prior distributions (mean = 0, std. dev. = 5) and the scale parameters of the hierarchical choice attribute effects were assigned weakly informative Cauchy prior distributions (mean = 0, scale = 2.5; Gelman et al. 2014). The model included a full covariance matrix, modelling correlations between choice attribute effects which was given an uninformative LKJ prior (shape parameter, η = 1; Stan Development Team 2015)

*Model fitting*

MCMC sampling was carried out in Stan which uses Hamiltonian Monte Carlo (Stan Development Team 2015). For each model four chains were run in parallel. The first 2000 iterations were discarded as a burn-in, during which time convergence was invariably reached. Subsequently the chains were run for a further 12,500 iterations per chain, thinned by 10 to give a combined final sample of 5,000 iterations per model. Convergence was assessed using Gelman-Rubin statistics, with values <=1.01 taking to indicate adequate convergence, and visually using trace plots (Gelman et al. 2014). Effective sample size and Monte Carlo Standard Error (MCSE) were checked as measures of the adequacy of MCMC chain length (Gelman et al. 2014). Posterior predictive checks were also conducted and visually examined to assess whether the model provided a good fit to the data and correctly predicted the proportions chosen overall and for each choice situation (Gelman et al. 2014).

*Model interpretation*

The coefficient estimates for each of the choice attributes in the fitted model represent estimates of marginal utility (i.e. the change in the level of wellbeing experienced for a unit increase in the attribute relative to the baseline level; Louviere et al. 2000). Positive coefficient estimates indicate that increasing levels of an attribute lead to increased wellbeing and larger coefficient estimates indicate that changes in the attribute have a larger effect on wellbeing. The ratio of two parameter estimates can also be interpreted as the average rate of substitution between attributes (i.e. the amount of one livelihood component that an average individual in the population would be willing to give up in return for a specified quantity of another livelihood component).

Aizaki, H., T. Nakatani, and K. Sato 2014. Stated preference methods using R. CRC Press.

Gelman, A. 2008. Scaling regression inputs by dividing by two standard deviations. Statistics in medicine **27**:2865-2873.

Gelman, A., J. B. Carlin, H. S. Stern, D. B. Dunson, A. Vehtari, and D. B. Rubin 2014. Bayesian Data Analysis. Taylor & Francis, CRC Press, Boca Raton, Florida.

Hoyos, D. 2010. The state of the art of environmental valuation with discrete choice experiments. Ecological Economics **69**:1595-1603.

Louviere, J. J., D. A. Hensher, and J. D. Swait 2000. Stated choice methods: analysis and applications. Cambridge University Press.

Nielsen, M. R., J. B. Jacobsen, and B. J. Thorsen. 2014. Factors Determining the Choice of Hunting and Trading Bushmeat in the Kilombero Valley, Tanzania. Conservation Biology **28**:382-391.

Plummer, M., N. Best, K. Cowles, and K. Vines. 2006. CODA: Convergence Diagnosis and Output Analysis for MCMC. R News **6**:7-11.

Stan Development Team. 2015. Stan Modeling Language Users Guide and Reference Manual, Version 2.6.0.
