# Supplementary material for: Gender Differentiated Preferences for a Community-Based Conservation Initiative
Source: PLoS One. 2016 Mar 29;11(3):e0152432. doi: 10.1371/journal.pone.0152432 (PMC4811562; doi:10.1371/journal.pone.0152432)
Supplement: S1 Table — Age sets are groupings of individuals of similar ages with distinct social and cultural roles and responsibilities (see [22, 30] for a full description). Within our sample the age ranges of the men in each age set were: Ilmeshuki, 18–38; Ilmejooli, 23–45; Ilkishili, 35–52; Ilkitoip/Iseuri, 48–74. Married women take on the age set of their husbands, so are commonly younger than their age set would suggest. The age ranges for women associated with each age set were: Ilmeshuki, 16–32; Ilmejooli, 16–38; Ilkishili, 18–45; Ilkitoip/Iseuri, 16–70. (DOCX) [file pone.0152432.s006.docx]

|  | **Male** | | **Female** | |
| --- | --- | --- | --- | --- |
|  | **Members** | **Non-members** | **Members** | **Non-members** |
| *N* | 89 | 100 | 150 | 49 |
| *Age (median; years)* | 35 | 35 | 34 | 28 |
| *Age set: Ilmeshuki (%)* | 37.1% | 37.0% | 24.0% | 40.8% |
| *Age set: Ilmejooli (%)* | 24.7% | 28.0% | 9.3% | 12.2% |
| *Age set: Ilkishili (%)* | 19.1% | 15.0% | 30.0% | 24.5% |
| *Age set:* *Ilkitoip or Iseuri (%)* | 19.1% | 20.0% | 36.7% | 22.4% |
| *Received any formal education? (% yes)* | 24.7% | 27.0% | 15.3% | 20.4% |
| *Time living in area*  *(median; years)* | 30 | 24 | 16 | 6 |
| *Household size (median; people)* | 9.0 | 9.5 | 6.0 | 5.0 |
| *Head of household? (% yes)* | 94.4% | 84.0% | 8.0% | 20.4% |
| *Community leadership position? (% yes)* | 22.5% | 16.0% | 39.3% | 18.4% |
| *Primary occupation*  *(% Pastoralist)* | 100% | 100% | 1.3% | 2.0% |
| *Primary occupation*  *(% House wife)* | 0% | 0% | 72.7% | 85.7% |
| *Primary occupation (% Other)* | 0% | 0% | 26.0% | 12.3% |
| *Cattle (% owning more than 51)* | 52.9% | 32.0% | 30.7% | 47.0% |
| *Small stock*  *(% owning more than 101)* | 63.0% | 43.0% | 12.6% | 40.8% |
| *Land owned (mean; acres)* | 150.9 | 79.4 | 120.9 | 70.6 |
| *Land inside a conservancy (mean; acres)* | 103.0 | 3.1 | 117.9 | 0.0 |
| *Traditional buildings (median)* | 1 | 1 | 1 | 1 |
| *Iron roofed buildings (median)* | 1 | 1 | 1 | 1 |
| *Owns any vehicles (% yes)* | 28.1% | 15.0% | 29.3% | 36.7% |
